# Satisfactory Evaluation of Call Service Using AI After Ureteral Stent Insertion: Randomized Controlled Trial

**DOI:** 10.2196/56039

**Published:** 2025-01-21

**Authors:** Ukrae Cho, Yong Nam Gwon, Seung Ryong Chong, Ji Yeon Han, Do Kyung Kim, Seung Whan Doo, Won Jae Yang, Kyeongmin Kim, Sung Ryul Shim, Jaehun Jung, Jae Heon Kim

**Affiliations:** 1 AI product Biz Team, AI service division, SK Telecom Seoul Republic of Korea; 2 College of Business, KAIST Seoul Republic of Korea; 3 Department of Urology Soonchunhyang University Seoul Hospital Soonchunhyang University Medical College Seoul Republic of Korea; 4 Social Safety Net Team, ESG Office, SK Telecom Seoul Republic of Korea; 5 Department of Engineering University of Hong Kong Hong Kong China (Hong Kong); 6 Department of Biomedical Informatics College of Medicine Konyang University Daejeon Republic of Korea; 7 Department of Preventive Medicine Korea University College of Medicine Seoul Republic of Korea

**Keywords:** artificial intelligence, AI, ureteral stent, complications, randomized controlled trial, urologic procedures, urology, patients, information resources

## Abstract

**Background:**

Ureteral stents, such as double-J stents, have become indispensable in urologic procedures but are associated with complications like hematuria and pain. While the advancement of artificial intelligence (AI) technology has led to its increasing application in the health sector, AI has not been used to provide information on potential complications and to facilitate subsequent measures in the event of such complications.

**Objective:**

This study aimed to assess the effectiveness of an AI-based prediction tool in providing patients with information about potential complications from ureteroscopy and ureteric stent placement and indicating the need for early additional therapy.

**Methods:**

Overall, 28 patients (aged 20-70 years) who underwent ureteral stent insertion for the first time without a history of psychological illness were consecutively included. A “reassurance-call” service was set up to equip patients with details about the procedure and postprocedure care, to monitor for complications and their severity. Patients were randomly allocated into 2 groups, reassurance-call by AI (group 1) and reassurance-call by humans (group 2). The primary outcome was the level of satisfaction with the reassurance-call service itself, measured using a Likert scale. Secondary outcomes included satisfaction with the AI-assisted reassurance-call service, also measured using a Likert scale, and the level of satisfaction (Likert scale and Visual Analogue Scale [VAS]) and anxiety (State-Trait Anxiety Inventory and VAS) related to managing complications for both groups.

**Results:**

Of the 28 recruited patients (14 in each group), 1 patient in group 2 dropped out. Baseline characteristics of patients showed no significant differences (all *P*>.05). Satisfaction with reassurance-call averaged 4.14 (SD 0.66; group 1) and 4.54 (SD 0.52; group 2), with no significant difference between AI and humans (*P*=.11). AI-assisted reassurance-call satisfaction averaged 3.43 (SD 0.94). Satisfaction about the management of complications using the Likert scale averaged 3.79 (SD 0.70) and 4.23 (SD 0.83), respectively, showing no significant difference (*P*=.14), but a significant difference was observed when using the VAS (*P*=.01), with 6.64 (SD 2.13) in group 1 and 8.69 (SD 1.80) in group 2. Anxiety about complications using the State-Trait Anxiety Inventory averaged 36.43 (SD 9.17) and 39.23 (SD 8.51; *P*=.33), while anxiety assessed with VAS averaged 4.86 (SD 2.28) and 3.46 (SD 3.38; *P*=.18), respectively, showing no significant differences. Multiple regression analysis was performed on all outcomes, and humans showed superior satisfaction than AI in the management of complications. Otherwise, most of the other variables showed no significant differences (*P*.>05).

**Conclusions:**

This is the first study to use AI for patient reassurance regarding complications after ureteric stent placement. The study found that patients were similarly satisfied for reassurance calls conducted by AI or humans. Further research in larger populations is warranted to confirm these findings.

**Trial Registration:**

Clinical Research Information System KCT0008062; https://tinyurl.com/4s8725w2

## Introduction

The initial stents designed for use in the ureter were the double-J stent and single pigtail stent [1-3]. Following their innovation, ureteric stents have become an essential component of urologic procedures; their applications include safeguarding against renal obstruction caused by stone fragment residues, edema, or blood clots; preventing the extravasation of urine; and for analgesia [4,5].

A number of pharmaceutical agents have been used in order to decrease the complications associated with ureteric stents and assessed in randomized controlled trials (RCTs). These include α-blockers, anticholinergic drugs, β-3 agonists, pregabalin, phosphodiesterase-5 inhibitors, antispasmodics, analgesics, anti-inflammatory drugs, and botulinum A toxin [6]. No studies have been carried out in order to predict the likely complications that may occur and to provide patients with this information, to offer individuals receiving stents reassurance, or to ensure that they receive timely follow-up appointments for any additional therapy required.

The concept of artificial intelligence (AI) was introduced during the 1950s, referring to the potential of machines to demonstrate human intelligence traits, such as being able to learn, reason, and solve problems [7]. The application of AI within health systems has now become a reality, owing to the rapid growth of computational resources, diminished data storage expenses, advanced complexity of algorithms, and widely available electronic health data [8-10]. Instruments facilitated by AI have made positive contributions in a number of health contexts to diagnostic work [11] and treatments [12], as well as to the provision of prognostic information, for example, mortality rates [13]. In renal stone pathologies, most researchers have evaluated the use of AI in terms of the precision of the diagnosis or in order to forecast the clinical results of therapy. The use of AI has infrequently been used in order to provide patients with data, before a ureteric stent intervention, regarding the possible complications that may arise and whether they would benefit from prompt adjunctive therapy.

In order to examine the efficacy of an AI-based prediction that could potentially provide patients with reassurance regarding the complications occurring in association with lithotomy and ureteric stent placement performed through ureteroscopy and to indicate the need for early additional therapy following the intervention, a pilot RCT was carried out. This is the initial study to investigate this topic within this clinical discipline.

## Methods

### Study Population

From October 2022 to December 2023, a total of 28 patients who underwent ureteral stent insertion with ureteroscopic ureterolithotomy were consecutively included in this single-institution study at the Department of Urology, Soonchunhyang University Hospital, Seoul, South Korea.

Patients were approached during their preoperative consultations and informed about the study. Patients who underwent ureteral stent insertion with ureteroscopic ureterolithotomy, patients who received intervention about urinary stone for the first time, patients who can communicate and are aged between 20 and <70 years, patients without history of psychologic illness, and patients without cognitive problems were included. Patients who were judged to be unsuitable for participation in the study, patients with cognitive impairment, and patients who had undergone the same procedure in the past were excluded. Korean version of the Alzheimer Disease-8, a brief informant-based measure designed to distinguish individuals with very mild dementia and mild cognitive impairment from those with normal cognition, was performed on all patients. Patients with cognitive impairment who had not been previously diagnosed and those with a score of 2 or higher were excluded from the study.

### Study Procedure

A call service named “reassurance-call” had been established to provide patients with information about the procedure and postprocedure care, to check for the presence and severity of complications, and to guide future treatments in case of complications. The algorithm of the reassurance-call can be found in Multimedia Appendix 1.

The included 28 patients who underwent ureteroscopic ureteral stent insertion were divided into 2 groups of 14 people each [14]. The power was determined to be 14, considering a dropout rate of 10% based on the minimum target number of 12 to confirm significance [15]. The 2 groups were reassurance-call service group by AI (group 1) and reassurance-call service group by humans (group 2). Allocation was performed with random permutation. JHK generated the random allocation sequence, YNG enrolled participants, and JYH randomly assigned patients into 2 groups. A complete list of patient characteristics can be found in Multimedia Appendix 2.

Operation agreement and cognitive questionnaire were obtained before the surgery. When patients were discharged, α-blockers and anticholinergics were prescribed to relieve irritation of the ureteral stent, and painkillers were prescribed as needed. On the day after discharge and on the 8th day, group 1 or 2 were randomly assigned and provided reassurance-call. Satisfaction with reassurance-call service was assessed on the 14th day after discharge.

**“**Reassurance-call” is a voice recognition-based AI service that relies on natural language processing technology, rather than a large language model, to reduce hallucinations and conduct surveys accurately. It leverages natural language understanding (NLU) technology to comprehend user intent and uses dialogue management techniques to facilitate smooth communication with patients. The “reassurance-call” service is trained in the following ways: voice recognition training, NLU training, and dialogue management training. First, the “reassurance-call” service improves its ability to recognize patients’ voices through voice recognition training. This process involves analyzing patients’ voices to update the voice recognition model. In the pilot trial using “reassurance-call,” accurate recognition of numbers was crucial due to the use of the Likert scale. SK Telecom, with experience in providing voice-based services like Tmap (navigation; Tmap Mobility) and Btv (media; SK Broadband), brought expertise in number recognition. However, for this pilot, additional training and tuning were conducted using voices from various age groups and genders. Second, the “reassurance-call” service enhances its ability to understand patients’ intentions through NLU training. This involves analyzing patients’ speech to update the NLU model. During the pilot trial, additional intent lists were defined and trained to accurately handle responses when patients answered with text rather than numbers (eg, if the first option for a question about pain frequency was “often,” and the patient responded with “often,” “first one,” etc, instead of “1”). Third, the “reassurance-call” service improves its ability to manage conversations with patients through dialogue management training. This involves understanding the patient’s intent to provide appropriate responses. In the pilot trial, scenarios were additionally defined for situations where patients wanted to revise a previously answered question, and the dialogue algorithm was trained to handle these scenarios smoothly.

### Statistics

The primary outcome was satisfaction with reassurance-call service assessed with a 5-point Likert scale. Secondary outcomes were satisfaction with how complications were handled and anxiety about complications. Satisfaction was scored with a 5-point Likert scale and 10-point Visual Analogue Scale (VAS), and anxiety was assessed with the State-Trait Anxiety Inventory (STAI), which is a 20-item self-report measure of anxiety using a 4-point Likert-type for each item, and the VAS.

Continuous variables were represented by mean and SD, and categorical variables were represented by frequency and percentage. We performed chi-square tests and *t* tests to confirm no significant selection bias at baseline between AI (group 1) and humans (group 2). To identify the variables that affected between 2 groups, we first examined each outcome (*reassurance-call by AI satisfaction.Likert*, *Complication.satisfaction.Likert*, *Complication.satisfaction.VAS*, *Complication.anxiety.STAI*, and *Complication.anxiety.VAS*) using *t* tests. Due to the small sample size per group, nonparametric tests (Mann-Whitney *U* test) were also conducted. Multiple regression analysis was then performed to identify the factors associated with satisfaction and anxiety outcomes. The significance of multicollinearity was assessed by comparing the variance inflation factors between independent variables. Statistical analysis was performed using R version 4.2.1 (The R Foundation for Statistical Computing). All statistics were 2-tailed, and *P* values <.05 were considered significant. In addition, the CONSORT-EHEALTH checklist can be found in Multimedia Appendix 3.

### Ethical Considerations

This study underwent a thorough ethical review and was approved by the institutional review board of Soonchunhyang University Hospital (IRB No. SCHUH 2022-07-013-001). Written informed consent was obtained from all patients, and the study data were deidentified. Participants received no compensation for their involvement in the study, and no participant images were uploaded.

## Results

**Figure 1:**
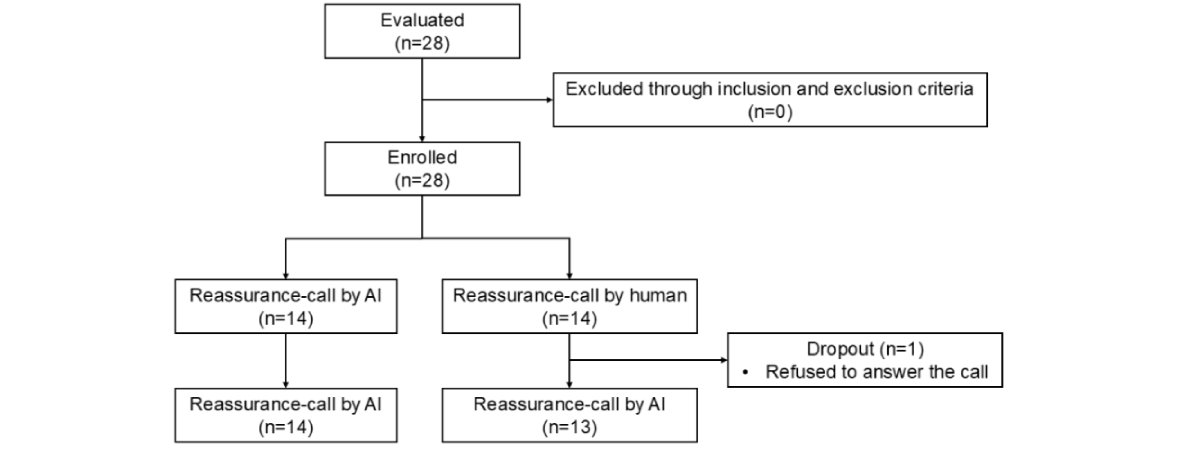
CONSORT (Consolidated Standards of Reporting Trials) flow diagram of patients selection. AI: artificial intelligence.

In total, 28 patients gave informed consent and were enrolled in the study. The study population was randomly assigned into 2 groups. One patient in group 2 was excluded because he refused to answer the reassurance-call after the first call (Figure 1). Baseline characteristics of patients are shown in Table 1. The mean height and weight of group 1 was 167.38 cm and 67.86 kg, while group 2 had a mean height of 166.81 cm and weight of 69.56 kg, respectively. Also, mean age was 55.14 years and 53.64 years, respectively. Among 14 patients in group 1, a total of 10 were male and 4 were female, while 8 were male and 6 were female in group 2. In group 1, the upper ureter stone was the most common (5/14, 36%), and in group 2, the distal ureter stone was the most common location of urinary stone (7/14, 50%). Mean stone size was 5.12 mm in group 1 and 6.26 mm in group 2, but grade-1 hydronephrosis was the most common in both groups (11/14, 79% and 12/14, 86%, respectively). In both groups, procedures were usually finished with a semirigid ureteroscope (9/14, 64% in both groups), and only 1 patient in group 2 underwent PCNL with a nephroscope. All the characteristics were statistically insignificant (all *P*>.05).

**Figure 2:**
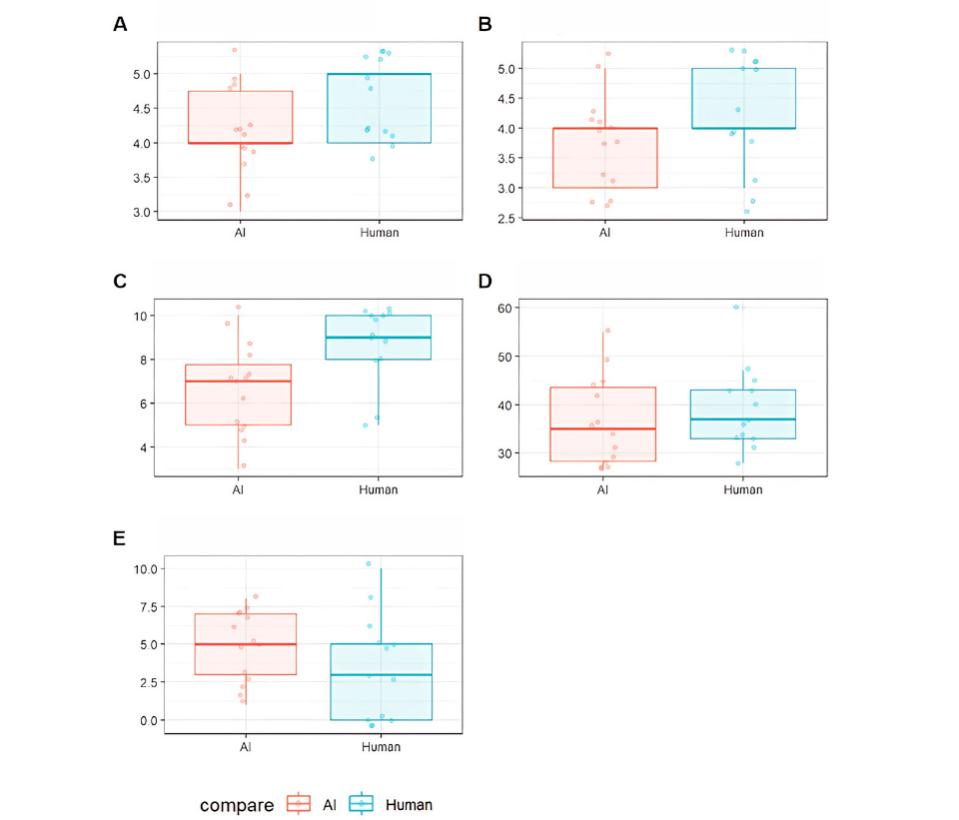
Box plots for satisfaction or anxiety after the reassurance-call service: (A) reassurance-call by AI satisfaction.Likert, (B) Complication.satisfaction.Likert, (C) Complication.satisfaction.VAS, (D) Complication.anxiety.STAI, and (E) Complication.anxiety.VAS. AI: artificial intelligence; STAI: State-Trait Anxiety Inventory; VAS: Visual Analogue Scale.

Primary and secondary outcomes are presented in Table 2. Satisfaction of reassurance-call showed a mean of 4.14 (SD 0.66) in group 1 and a mean of 4.54 (SD 0.52) in group 2. There was no significant difference between satisfaction for reassurance-call by AI and humans (*P*=.11). Satisfaction for reassurance-call by AI showed a mean of 3.43 (SD 0.94). Satisfaction about the management of complications using the Likert scale scored a mean of 3.79 (SD 0.70) in group 1 and a mean of 4.23 (SD 0.83) in group 2, which was not significantly different (*P*=.14), but it showed a significant difference when using VAS (*P*=.01), with a mean of 6.64 (SD 2.13) in group 1 and mean 8.69 (SD 1.80) in group 2. Anxiety about complications using STAI scored a mean of 36.43 (SD 9.17) in group 1 and a mean of 39.23 (SD 8.51) in group 2, and anxiety assessed with VAS scored a mean of 4.86 (SD 2.28) in group 1 and a mean of 3.46 (SD 3.38) in group 2. There were no significant differences (*P*=.33 and *P*=.18, respectively). The outcomes were presented using box plots in Figure 2.

Multiple regression analysis was performed on all outcomes and presented in Multimedia Appendix 4. Regarding satisfaction with the reassurance-call service using the Likert scale, there was no variable that showed a significant difference (all *P*>.05). However, in the analysis about satisfaction for management of complications using the Likert scale, reassurance-call by AI showed significantly lower satisfaction than reassurance-call by humans (*P*=.03), and satisfaction significantly increased as stone size increased (*P*=.03). Similarly, in the analysis about satisfaction for the management of complications using the VAS, reassurance-call by AI showed significantly lower satisfaction than reassurance-call by humans (*P*=.02). There was no variable that showed a significant difference in the analysis of anxiety for complications (all *P*>.05).

**Table 1:** Baseline characteristics of the patients.

Characteristics			Artificial intelligence (n=14)		Human (n=14)		*P* value
**Height (cm), mean (SD)**			167.38 (11.27)		166.81 (8.77)		.88
**Weight (kg), mean (SD)**			67.86 (13.02)		69.56 (14.61)		.75
**Age (years), mean (SD)**			55.14 (6.69)		53.64 (12.98)		—^a^
**Sex, male, n (%)**			10 (71)		8 (57)		.70
**Stone site, n (%)**							.25
	Lower ureter	4 (29)		7 (50)			
	Mid ureter	4 (29)		2 (14)			
	Upper ureter	5 (36)		3 (21)			
	Renal pelvis	1 (7)		2 (14)			
**Stone size (mm), mean (SD)**			5.12 (1.71)		6.26 (3.55)		.29
**Hydronephrosis, n (%)**							>.99
	Grade 0	2 (14)		1 (7)			
	Grade 1	11 (79)		12 (86)			
	Grade 2	1 (7)		1 (7)			
**Procedure, n (%)**							>.99
	Flexible ureteroscope	5 (36)		4 (29)			
	Semi-rigid ureteroscope	9 (64)		9 (64)			
	Nephroscope	0 (0)		1 (7)			

^a^Not applicable.

**Table 2:** Satisfaction or anxiety after reassurance-call service.

	AI^a^	Human	*P* value (nonparametric)
Reassurance-call satisfaction.Likert, mean (SD)	4.14 (0.66)	4.54 (0.52)	.11
Reassurance-call by AI satisfaction.Likert, mean (SD)	3.43 (0.94)	—^b^	—
Complication.satisfaction.Likert, mean (SD)	3.79 (0.70)	4.23 (0.83)	.14
Complication.satisfaction.VAS^c^, mean (SD)	6.64 (2.13)	8.69 (1.80)	.01
Complication.anxiety.STAI^d^, mean (SD)	36.43 (9.17)	39.23 (8.51)	.33
Complication.anxiety.VAS, mean (SD)	4.86 (2.28)	3.46 (3.38)	.18

^a^AI: artificial intelligence.

^b^Not applicable.

^c^VAS: Visual Analogue Scale.

^d^STAI: State-Trait Anxiety Inventory.

## Discussion

### Principal Findings

Hypothetically, a reassurance call generated by AI within a specific clinical context may provide patients with some knowledge regarding the expected complications following the insertion of a ureteric stent and therefore make them more satisfied with the outcome of the procedure. As anticipated, in some instances, human monitoring calls provided a higher satisfaction rating, but in general, it was established that safety data provided by an AI-based intervention could be used to substitute information provided by humans. This is the first research to demonstrate this outcome.

In our study, when comparing group 1 (AI) and group 2 (human), patients were more satisfied with calls made by a human than those made by AI. However, group 1 also received a score exceeding 4 out of 5, achieving a sufficiently high score on the Likert scale. Regarding complications, group 2 showed a significantly higher satisfaction score by the VAS than group 1; however, there was no significant difference between the 2 groups by other measurement tools including Likert-scale anxiety scores. This implies the possibility that AI can replace human calls to some extent.

Discomfort and the presence of blood in the urine are the most frequently occurring side effects following ureteric stent insertion, both of which patients find worrisome and may induce anxiety. The evidence base suggests that symptoms arising in this situation should be approached using combination therapy, for example, α-blockers, anticholinergic agents, anti-inflammatory drugs, and opiates. The rationale underlying the symptoms of loin and suprapubic discomfort, and urine-related issues, for example, frequency, urgency, incontinence, or pain, is not entirely clear. It has been proposed that the stent may act as a mechanical irritant, and that reflux may generate raised pressures within the urinary tract. These issues have not been resolved by either stent design modifications or the administration of pharmaceutical agents either during or following stent insertion. It is essential from the patient’s perspective to administer the appropriate medications to address likely complications following the procedure, as well as to be able to provide them with reassurance regarding their symptoms and to monitor for more serious adverse events.

Patient care quality can be enhanced by the application of AI, which enables algorithms pertaining to diagnosis and therapy to be generated for use in patients with renal lithiasis. To date, AI has been used to determine the constituents of renal stones with some precision, to support a diagnosis of lithiasis, and to forecast clinical endpoints following operative interventions [16]. There are, however, further aspects of this clinical domain that have the potential to be assisted by AI.

The widespread use of tools based on AI in routine clinical work has been described in a recent publication by Lam et al [17], with applications identified in 13 clinical disciplines. The majority of relevant RCTs were performed in gastroenterology; 15 studies examined the use of AI to support endoscopic procedures.

Instruments incorporating biosignal-based AI were used in a high proportion of RCTs. In a study of patients with malignancy, those individuals offered a de novo smartphone intervention based on AI demonstrated lower pain scores and were less likely to have a negative mindset toward oncotherapy than control subjects [18].

In some cases, operative procedures for renal stones can be carried out as day cases or in an outpatient facility. In this setting, there should be a consensus on how care is offered to patients who develop postoperative issues. Typically, this is provided by making telephone contact with the patient on the day after the procedure in order to monitor their recovery process and to establish whether they are experiencing any complications [19]. The number of patients requiring same-day procedures, however, is increasing, and this method of follow-up is labor-intensive and takes time that could otherwise be used for clinical patient care. The French Society of Anesthesia and Intensive Care is conducting a study (OPERA) throughout France in order to determine the way in which ambulatory interventions are organized. They established that no calls are made to approximately 15% of patients; this figure may be as high as 55% on Saturday mornings [20]. The majority of follow-up calls, that is, 70%, are made by nursing staff; secretaries and doctors carry out 5% and 1%, respectively. These statistics therefore provide an incentive to develop automated systems to review patients. It is essential that patients remain satisfied with the use of AI, as opposed to a human, to assess their safety, as demonstrated in this study. This is a more advanced system than automated text messages and has the ability to converse with patients.

Although our study is the first to show the potential for safe calls using AI, there are some limitations. The first is that, even if AI is introduced, it is not in the form of conversation. A conversation was had with the patient, during which their symptoms were assessed according to a predetermined script, regardless of whether this was carried out by a human or an AI-based tool. Generative AI was not used in this study, and so, as expected, patients felt less satisfied than if they had conversed with a human. Generative AI is not feasible at present in clinical medicine, but despite this potential shortfall, the outcome of the study was extremely promising. A point was made of specifically enquiring about symptoms of major complications, for example, difficulty passing urine, evident hematuria, and marked pyrexia, and to note if these were absent. A further benefit of this study was that patients could be reassured regarding any potential complications that were arising. We checked for complications by directly asking patients about predictable important complications, such as high fever, severe pain, and hematuria, and guided them to return to the hospital in cases of high fever, so that serious urinary tract infections could be treated early.

### Conclusions

Through our study, we have confirmed for the first time that AI can replace humans in identifying complications that occur after ureteral stent insertion and can also play a role in alleviating anxiety. Although this pilot study included only a small number of participants, the outcomes demonstrated similar satisfaction rates among patients regardless of whether they received a follow-up call made by the tool based on AI or a human. Additional prospective research is therefore merited in larger populations in order to substantiate these findings.

## Appendix

Multimedia Appendix 1 The algorithm of Ansim-call.

Multimedia Appendix 2 A complete list of patient characteristics.

Multimedia Appendix 3 CONSORT-eHEALTH V1.6.

Multimedia Appendix 4 Regression analysis for identifying risk factors.

## Abbreviations

AI: artificial intelligence
NLU: natural language understanding
RCT: randomized controlled trial
STAI: State-Trait Anxiety Inventory
VAS: Visual Analogue Scale

## Data Availability

Datasets generated during and/or analyzed during this study are available from the corresponding author on reasonable request.
